# Hydrogenotrophic Methanogenesis Under Alkaline Conditions

**DOI:** 10.3389/fmicb.2020.614227

**Published:** 2020-12-03

**Authors:** Richard M. Wormald, Simon P. Rout, William Mayes, Helena Gomes, Paul N. Humphreys

**Affiliations:** ^1^Department of Biological and Geographical Sciences, University of Huddersfield, Huddersfield, United Kingdom; ^2^Department of Geography, Geology and Environment, University of Hull, Hull, United Kingdom; ^3^Food, Water, Waste Research Group, Faculty of Engineering, University of Nottingham, Nottingham, United Kingdom

**Keywords:** alkaliphiles, radioactive waste, hydrogenotrophic methanogens, acetoclastic methanogens, alkaline

## Abstract

A cement-based geological disposal facility (GDF) is one potential option for the disposal of intermediate level radioactive wastes. The presence of both organic and metallic materials within a GDF provides the opportunity for both acetoclastic and hydrogenotrophic methanogenesis. However, for these processes to proceed, they need to adapt to the alkaline environment generated by the cementitious materials employed in backfilling and construction. Within the present study, a range of alkaline and neutral pH sediments were investigated to determine the upper pH limit and the preferred route of methane generation. In all cases, the acetoclastic route did not proceed above pH 9.0, and the hydrogenotrophic route dominated methane generation under alkaline conditions. In some alkaline sediments, acetate metabolism was coupled to hydrogenotrophic methanogenesis *via* syntrophic acetate oxidation, which was confirmed through inhibition studies employing fluoromethane. The absence of acetoclastic methanogenesis at alkaline pH values (>pH 9.0) is attributed to the dominance of the acetate anion over the uncharged, undissociated acid. Under these conditions, acetoclastic methanogens require an active transport system to access their substrate. The data indicate that hydrogenotrophic methanogenesis is the dominant methanogenic pathway under alkaline conditions (>pH 9.0).

## Introduction

Globally, geological disposal is largely the preferred choice for the management of intermediate level radioactive wastes (ILWs; IAEA, 2011) with a number of countries pursuing the establishment of geological disposal facilities (GDFs; Faybishenko et al., 2016). The exact design of these facilities is dependent on the host geology; however, there are often a number of common features, including the use of cementitious construction and backfill materials, the presence of metal in the construction, container and waste components, and organic, primarily cellulose, containing wastes.

The presence of metallic and organic materials means that abiotic and biotic gas generating processes (NEA, 2014) are likely to proceed within a GDF resulting in gas generation being evaluated in a number of international waste management programs (Felicione et al., 2003; NDA, 2010; Avis et al., 2011; Summerling, 2013; Poller et al., 2016). The anaerobic corrosion of metals will generate molecular hydrogen (NDA, 2010; Libert et al., 2011), which can in turn, act as an electron donor for hydrogenotrophic methanogenesis (Libert et al., 2011; Bagnoud et al., 2016). The alkaline conditions generated by the cementitious materials will promote the alkaline hydrolysis of cellulosic wastes (Humphreys et al., 2010). This abiotic process generates a range of cellulose degradation products (CDPs; Rout et al., 2015b; Charles et al., 2019) collectively which are dominated (>70%) by isosaccharinic acids (ISAs; Almond et al., 2012).

Recent research has focused on the use of lime kiln waste sites to provide an insight into the biodegradation of CDP components under alkaline conditions (Milodowski et al., 2013; Charles et al., 2019). Despite the harsh geochemical environment (pH > 10.0), these sites support extensive and diverse bacterial and archaeal populations (Burke et al., 2012; Kyeremeh et al., 2016) capable of a wide range of metabolic and energy generating processes (Bassil et al., 2014; Rout et al., 2014, 2015a,b), including methanogenesis (Rout et al., 2015b). The information that is available for these sites suggests that the hydrogenotrophs dominate the methanogenic populations despite the availability of acetic acid and the absence of competition by other acetate consuming pathways (Charles et al., 2015; Rout et al., 2015b; Kyeremeh et al., 2016).

Given the importance of methanogenesis to gas generation within a GDF, a range of anthropogenic alkaline environments of various ages were investigated to determine the impact of environmental pH on the nature and extent of methanogenic activity.

## Materials and Methods

### Sampling Site Investigations

The investigation involved sediments from three lime kiln waste sites (≈25 to ≈150 years old; New Lime sites, designated B, H, and T; Charles et al., 2019), five field kiln sites (Johnson, 2008; 200–300 years old; Old Lime sites, designated LK1, LK2, LK3, LK4, and LK5), and four steel industry waste sites (5 to ≈30 years old; Mayes et al., 2008; Steel sites, designated CW, CS, RC, and SC; see Supporting Information). In addition, a neutral pH, freshwater anaerobic sediment was employed as a control (Control). In the case of the New Lime, Steel, and Control sites, hand cored samples (dia. 30 mm) were taken at depth (~1 m). In the case of the Old Lime sites, samples were taken from a shallow depth of 10–30 cm using a stainless steel trowel. All sampling equipment was surface sterilize using a commercial hypochlorous acid spray (Redditch Medical Ltd., Stockton on Tees, UK). Sterile plastic sample containers were filled to maximum capacity with sediments and where possible with the associated pore waters in order to minimize the presence of oxygen. The pH values of the associated pore waters were determined *in situ* with a hand-held pH probe (Mettler Toledo, Columbus, OH, Unites States) and the pH of the soil/sediment determined using standard methods (B.S.I, 2005).

### Microcosm Investigations

All microcosms employed in the study were incubated in the dark at 25°C, and the incubation time was dependent on the time taken for the microcosm to generate detectable methane and ranged from 2 to 8 weeks. Initial microcosms employing New Lime and Control sediments were established by mixing a soil/sediment sample (5 g) from each location with 5 ml of sterile, nitrogen purged mineral media (MM; Supplementary Tables S1, S2; B.S.I, 2005) adjusted to pH 10.0 with 4 M NaOH for New Lime microcosms and pH 7.0 for the Control microcosms. Slurries were then transferred to 100 ml crimp top bottles containing 100 ml of MM supplemented with 10% v/v CDP and a hydrogen:nitrogen (10:90) headspace (BOC Ltd., Guildford, UK). A CDP/hydrogen enrichment was chosen since this had previously been shown (Rout et al., 2014, 2015a; Kyeremeh et al., 2016) to support a diverse range of microbial processes and provided a source of fermentable substrates, acetate, and hydrogen. CDP was prepared as previously described (Rout et al., 2014, 2015a) All microcosm experiments were carried out in duplicate.

On the detection of methane initial enrichments were sub-cultured twice at the starting pH by inoculating sterile microcosms with 5 ml of the previous microcosm suspension (Supplementary Figure S1). This was carried out to reduce the impact of residual fermentable substrates, mineral components, and other electron acceptors [e.g., Iron (III) and sulphate] present within the inoculating sediment.

Once methane generation was detected at the starting pH, each sediment was sequentially exposed to a range of pH values ranging from 7.0 to 12.0 (Supplementary Figure S1) as described below. Every microcosm that was methane positive was sub-cultured at the next pH in the series and, hydrogen and acetate enrichments were created at the original pH (Supplementary Figure S1). The last pH in the series that failed to generate methane was still sub-cultured into hydrogen and acetate enrichments. These enrichments were performed by transferring 5 ml of the microcosm suspension to a 100 ml crimp top bottle containing 100 ml of MM with either a hydrogen: carbon dioxide: nitrogen (10:10:80; BOC Ltd., Guildford, United Kingdom) headspace or MM supplemented with Na-acetate (30 mM) with a 100% nitrogen headspace. A set of blank microcosms were also established as described above with MM, soil, and a 100% nitrogen atmosphere.

### Chemical Analysis

Headspace hydrogen and methane was determined *via* an Agilent GC6850 equipped with HP-PLOT/Q column with particle traps ((35 m × 0.32 mm × 20 μm), Agilent Technologies, Santa Clara, CA, United States) employing thermal conductivity detection. Liquid microcosm samples (1 ml) were also removed over the course of incubation using degassed syringes and needles. Acetic acid concentrations were determined using gas chromatography (Hewlett Packard Ltd., London, United Kingdom) with a flame Ionization detector (GC-FID) as described previously (Rout et al., 2015a). To determine the presence of acetoclastic methanogenesis the metabolic inhibitor methylflouride was employed at an initial concentration of 1% as described previously (Daebeler et al., 2013). Duplicate reactors without inhibitor addition were also prepared and served as a control. Methane produced from CO_2_ (m_CO_2__) was calculated from inhibited microcosms002C and acetate-derived methane was calculated from methane production in uninhibited microcosms (m_acetate_ = m_total_ − m_CO_2__).

### 16S rRNA Gene Sequencing

Total genomic DNA was extracted from both soil/sediment samples and methanogenic enrichment cultures using the PowerSoil DNA Isolation Kit (Qiagen, Manchester, UK). The enrichment cultures were sampled after 2 weeks of incubation by the centrifugation (10,000 × *g*) of the microcosm fluid to pellet the cells prior to DNA extraction. The V4 region of the 16S rRNA gene was then amplified by PCR using primers 341f and 805r (Takahashi et al., 2014). Next generation sequencing of PCR products was carried out by ChunLab (South Korea) using the Illumina MiSeq platform. Sequences were then paired and clustered into OTUs using DADA2 employing R3.2.5 (Callahan et al., 2016), before being identified using the MegaBLAST search strategies (Altschul et al., 1997).

## Results

### CDP Fed NEW Lime Microcosms

**Figure 1:**
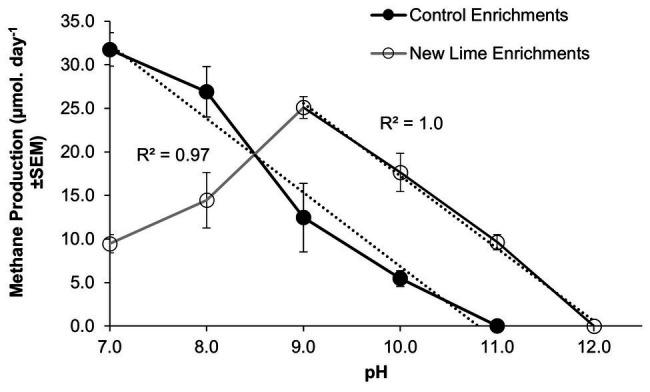
Methane production rates in CDP-fed microcosms employing Control and New Lime sediments operating between pH 7.0 and 12.0. Control microcosms (*n* = 2 at all pH values) had a pH optimum of 7.0 (*n* = 3), in contrast to the New Lime microcosms (*n* = 2 pH 7.0, *n* = 4 pH 8.0, *n* = 6 pH 9.0, *n* = 6 pH 10.0, and *n* = 2 pH 11.0) were optimal at pH 9.0. The upper pH limit for methanogenesis was pH 10.0 for the Control sediment, and this increased to pH 11.0 in the case of the New Lime sediments. No methane generation was detected at a pH >11.0 (“*n*” denotes the number of replicates analyzed).

Within microcosms utilizing CDP as a carbon source, the Control enrichments demonstrated a linear reduction in methane production between pH 7.0 and 11.0 (Figure 1), while the New Lime enrichments demonstrated an optimum pH of 9.0 for methane generation and a linear reduction down to pH 12.0. This optimum is below the *in-situ* pH of the New Lime sites (pH 11.5–13.0, Supplementary Table S3), suggesting that the *in-situ* populations have alternative strategies to manage these harsh pH values (Charles et al., 2017).

Mean methane production values for those microcosms producing methane are presented (Figure 1) in order to emphasize the overall trends observed. Across the pH range investigated the microbial populations enriched from the three New Lime sites demonstrated different responses to changing pH with the oldest site (site B; see Supplementary Material) generating methane across the whole range and the other two sites generating methane across narrower pH ranges (Supplementary Figure S2).

**Figure 2:**
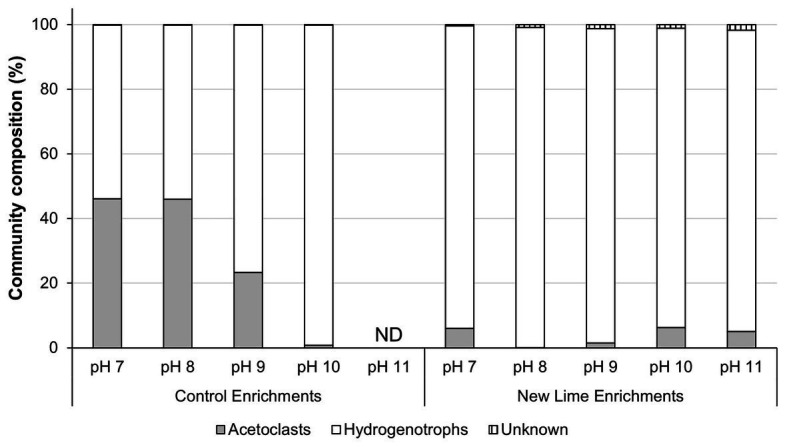
Methanogen communities in neutral and alkaline soil enrichments. When the neutral pH Control sediment was enriched with CDP the acetoclastic and hydrogenotrophic communities were present in roughly equal proportions at pH 7.0 and 8.0, respectively. As the pH increased to pH 10.0, the acetoclastic compostion fell as pH increased, with no methanogen community detectable at pH 11.0. In contrast the alkaline New Lime enrichments were dominated by hydrogenotrophic methanogens irrespective of enrichment pH (ND, none determined).

In the Control sediment enrichments, the archaeal community composition was composed of almost equal proportions of acetoclastic to hydrogenotrophic methanogens (46:54%; Figure 2) at pH 7.0 and 8.0. As the enrichment pH became more alkaline (pH ≥ 9.0), the community composition shifted toward a more hydrogenotrophic population Figure 2 even though acetate was present in these cultures. The New Lime sediment enrichments were dominated by hydrogenotrophic methanogens (Figure 2), suggesting that the long-term exposure to alkaline pH had selected against acetoclastic methanogens in these sediments.

### H_2_:CO_2_ and Acetate Fed New Lime microcosms

**Figure 3:**
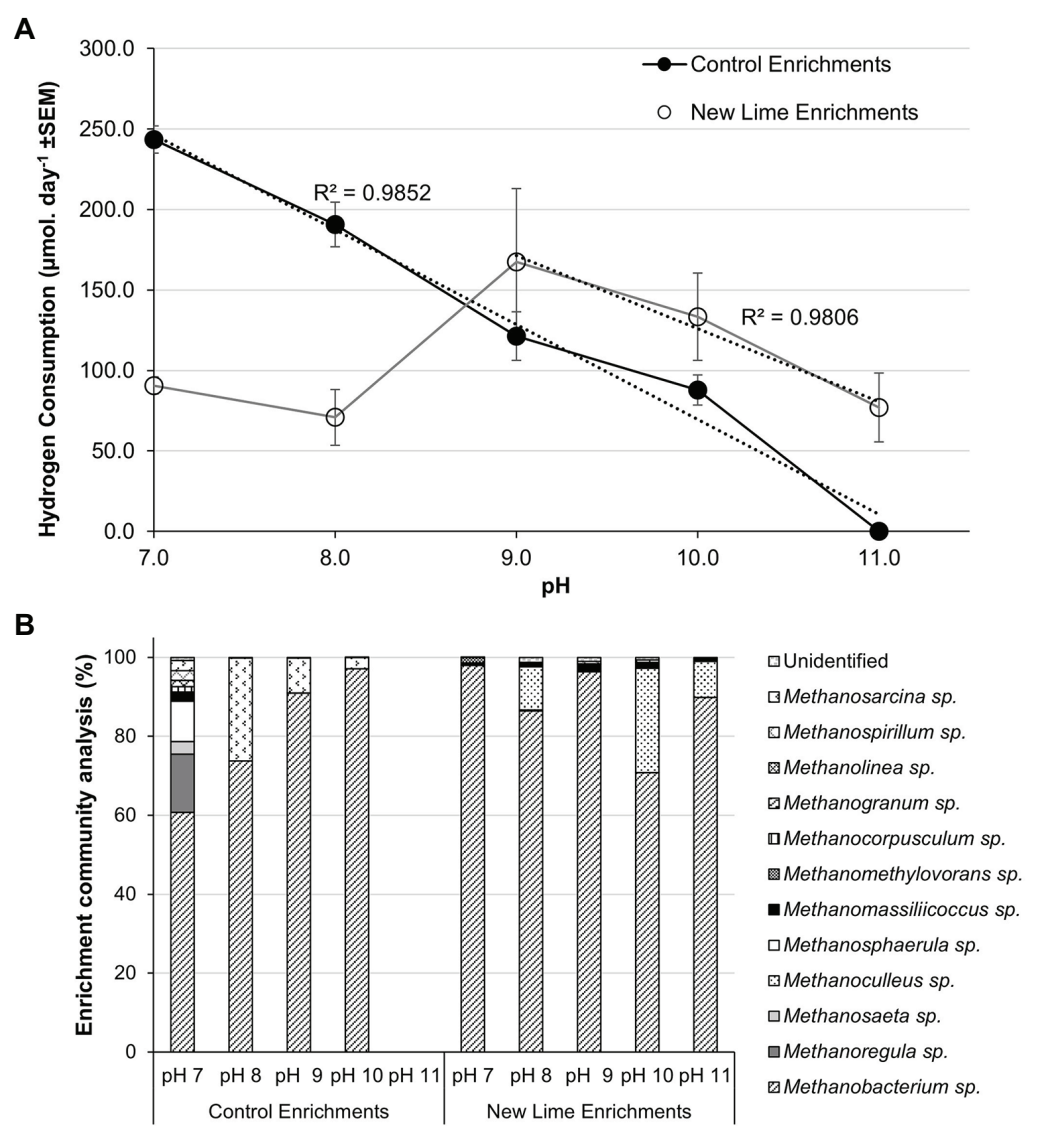
Hydrogen consumption rates **(A)** and methanogen populations **(B)** in H_2_/CO_2_ enriched microcosms from Control (*n* = 2) and New Lime (*n* = 6) soils. An increasing pH negatively impacted upon hydrogen consumption in Control soil enrichments (*n* = 2 at all pH values), while New Lime sediments (*n* = 4 pH 7.0, *n* = 4 pH 8.0, *n* = 6 pH 9.0, *n* = 6 pH 10.0, *n* = 4 pH 11.0) demonstrated an optimum pH of 9.0. Across all enrichments, *Methanobacterium* sp. were the dominant genus. ND, none detected (“*n*” denotes the number of replicates analyzed).

The trend toward hydrogenotrophic methanogenesis at alkaline pH was confirmed by the establishement of hydrogen and acetate enrichments from the CDP fed Control and New Lime enrichments at each pH. Within the H_2_:CO_2_ Control sediment enrichments, hydrogen consumption was at its highest at pH 7.0 at 243.3 μmol day^−1^, and became undetectable as pH increased to pH 11.0 (Figure 3A). The associated methanogenic communities were most diverse at pH 7.0 with 12 different methanogenic genera detected (Figure 3B). *Methanobacterium* spp. were the most dominant at this pH, comprising 60.7% of the community; with *Methanoregula* spp. (14.7%) and *Methanosphaerula* spp. (10.2%) also being prevalent within the community. As pH was increased through to pH 10.0, the loss of a number of the genera observed at pH 7.0 coincided with the further dominance of *Methanobacterium* spp., rising to 97.1% of the community at pH 10.0.

These findings were reinforced by the H_2_:CO_2_ New Lime enrichments in which *Methanobacterium* spp. were the dominant taxa detected across all pH (7.0–11.0) enrichments (Figure 3B). A negative relationship between hydrogen consumption and increasing pH was seen in these enrichments as pH decreased for pH 9.0–11.0, however hydrogen consumption was still detected at pH 11.0 (77.0 μmol day^−1^). The optimum pH for hydrogenotrophic methanogenesis was pH 9.0 with a hydrogen consumption of 167.5 μmol day^−1^. The alkaliphilic nature of the methanogenic population in the New Lime sediments was indicated by the fact that the rates of hydrogen metabolism decreased as the pH fell below pH 9.0 (Figure 3A). The data here reiterate that the optimum pH for hydrogenotrophic methanogenesis is pH 9.0 for communities of this type, and that it is not the availability of substrate from fermentation (such as CDP) that is a limiting factor. As was the case with the CDP reactors, not all the New Lime enrichments were active across the whole pH range (Supplementary Figure S3).

**Figure 4:**
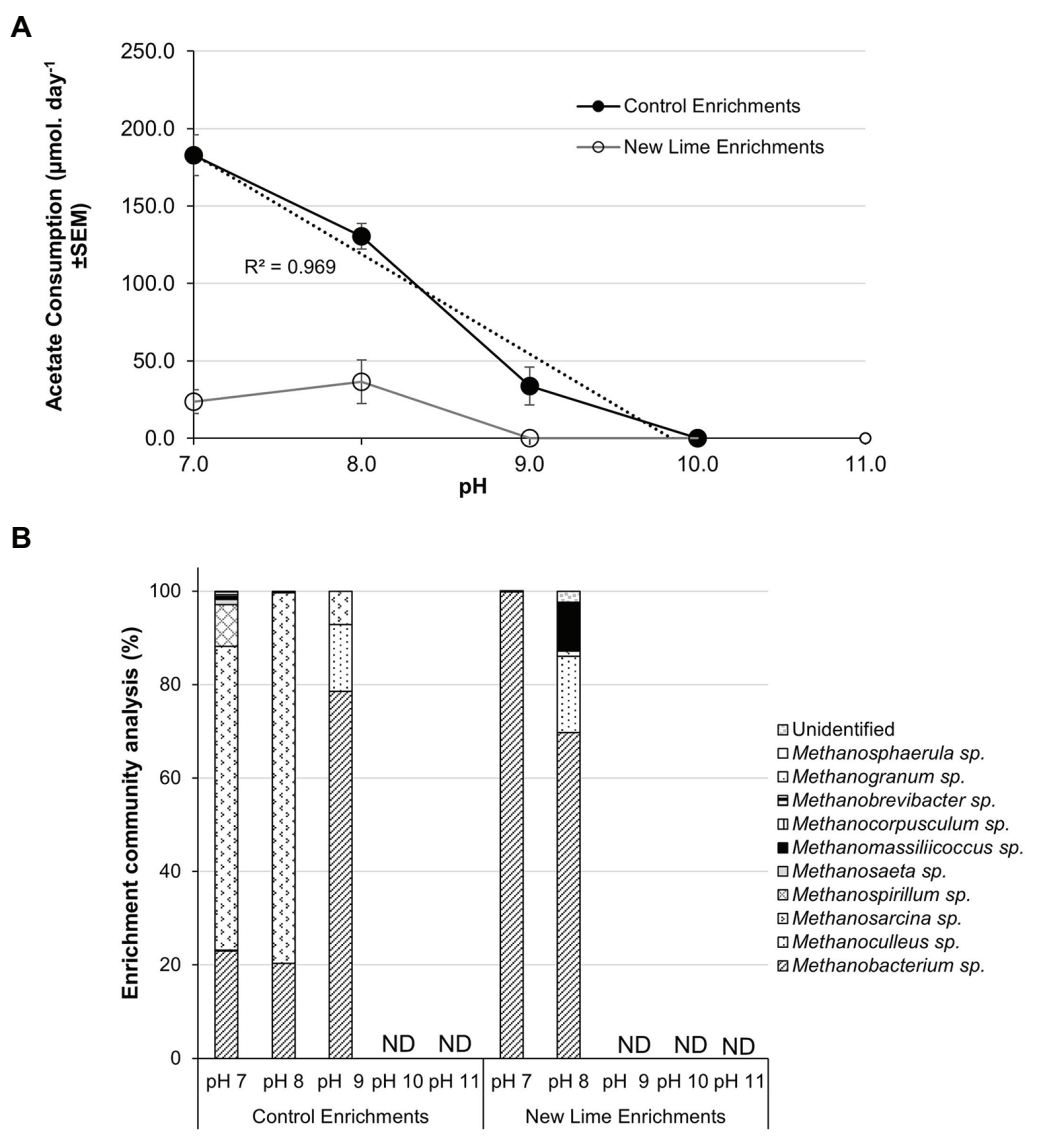
Acetate consumption rates **(A)** and methanogen populations **(B)** in sodium acetate enriched microcosms from Control and New lime soils. Increasing pH negatively impacted upon acetate consumption with no acetate consumption observed above pH 9.0 in either the Control (*n* = 2 at all pH values) enrichments or the New Lime enrichments (*n* = 2 pH 7.0, *n* = 2 pH 8.0). *Methanosarcina* spp. and *Methanobacterium* spp. were the dominant genera in Control enrichments while *Methanobacterium* sp. were dominant in the New Lime enrichments. ND, none detected (“*n*” denotes the number of replicates analyzed).

Acetate fed control enrichments contained both acetoclastic and hydrogenotrophic methanogens (Figure 4B). Hydrogenotrophic methanogens are able to generate methane from acetate *via* syntrophic acetate oxidation (SAO). SAO is an endergonic reaction with a Gibbs free energy of +104.6 kJ mol^−1^ and is, therefore, energetically less favorable than acetoclastic methanogenesis; however, when the oxidation of acetate is coupled to hydrogenotrophic methanogenesis, the total combined reaction is exergonic (ΔG0' = −31 kJ mol^−1^; Hattori, 2008). Few SAO bacteria have been described, but SAO is associated with the members of the phylum Firmicutes (Hattori et al., 2000; Balk et al., 2002; Westerholm et al., 2010; Manzoor et al., 2018). In the acetate fed Control enrichments (Figure 4A), a linear reduction (R^2^ = 0.97) in the rate of acetate removal was observed between pH 7.0 and 9.0 from 182.7 to 33.7 μmoles day^−1^; with a cessation of activity at pH 10.0. *Methanosarcina* spp. contributed 65.1% of the pH 7.0 and 79.3% of the pH 8.0 Control enrichment populations (Figure 4B). This fell to 7.1% within the pH 9.0 enrichment. The fall in acetate consumption between pH 7.0 and 8.0 coincided with the loss of both *Methanospirillium* and *Methanosaeta* spp. and a reduced proportion of *Methanosarcina* between pH 8.0 and 9.0. The loss of *Methanospirillium* spp. may suggest that SAO was active at pH 7.0 in these systems but was not able to adjust to the increase in pH.

Low levels of acetate consumption were only observed at both pH 8.0 (36.4 μmoles day^−1^) and pH 7.0 (23.6 μmoles day^−1^) in acetate enrichments from two of the three New Lime sites. However, the methanogenic populations in these enrichments were dominated by the hydrogenotrophic (Liu and Whitman, 2008; Dridi et al., 2012) *Methanobacterium*, *Methanoculleus*, and *Methanomassiliicossus* spp., with the potentially acetoclastic *Methanosarcina* spp. representing only 1.2% of the total archaeal populations at these pH values (Figure 4B). However, the members of the phylum Firmicutes which comprised 30–40% of the total populations of the pH 8.0 and 7.0 New Lime acetate enrichments, suggesting that SAO may be responsible for the low levels of acetate removal in these enrichments.

### Inhibition Studies

To investigate the role of in the acetate fed enrichments, the pH 7.0 acetate fed New Lime enrichments were sub-cultured in the presence of acetate and fluoromethane, an inhibitor of acetoclastic methanogenesis (Agneessens et al., 2018). Within these enrichments (Supplementary Figure S4), acetate removal was observed in both the presence and absence of inhibitor (removal rates: presence: 4.1 × 10^−2^ ± 4 × 10^−3^ mmol day^−1^; absence: 3.5 × 10^−2^ ± 4 × 10^−3^ mmol day^−1^), suggesting that acetate removal in these sediments was SAO linked. The addition of fluoromethane to pH 7.0 acetate fed Control enrichment saw a cessation of acetate removal, indicating that in this case, acetoclastic methanogens was the dominant acetate removal process even though the presence of hydrogenotrophs such as *Methanospirillium* spp. may suggest some SAO activities (Supplementary Figure S5). These results suggest that the role of SAO in methanogen generation in alkaline environments warrants further study.

### Steel and Old Lime Microcosms at pH 7 and 10

**Figure 5:**
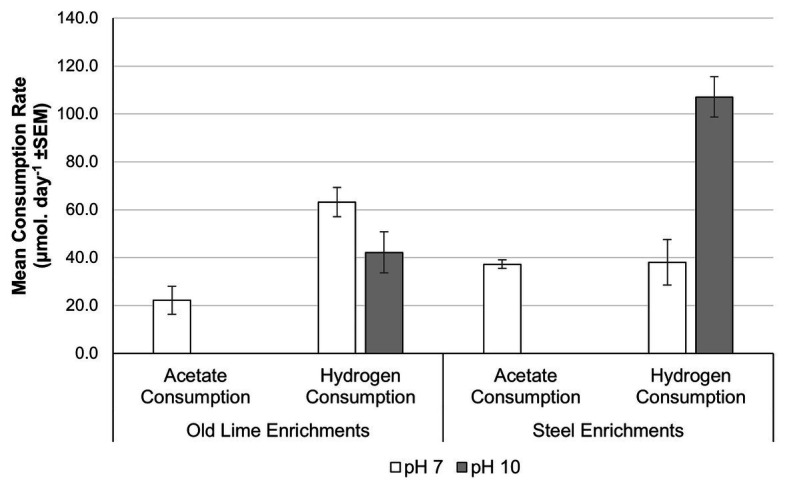
Acetate and hydrogen consumption from microcosms prepared using Old Lime and Steel site sediments as an enrichment inoculum. The pattern observed in the Old Lime (*n* = 10) and Steel (*n* = 8) sediments mirrored that seen with the New Lime and Control sediments with acetoclastic methanogenesis absent at alkaline pH (“*n*” denotes the number of replicates analyzed).

In order to determine the extent to which alkaline pH impacts upon acetoclastic methanogenesis across a wider selection of anthropogenic alkaline environments with more diverse methanogenic populations, sediments from the Steel and Old Lime sites were used to establish microcosms at pH 7.0 and 10.0. The Old Lime sediments (*n* = 5) had *in-situ* pH values closer to circum-neutral pH values, suggesting that the bulk environment had recovered from exposure to alkaline contamination. However, lime fragments are likely to be present within these sediments, suggesting that alkaline microsites will persist. The enrichments from these sediments were similar to the Control sediments, with both acetate and hydrogen consumptions being negatively impacted by an increase in pH from pH 7.0 to 10.0 (Figure 5). In particular, no acetate consumption or methane production was observed at pH 10.0. The acetate consumption rates at pH 7.0 were 2.0 × 10^−2^ (±7.7 × 10^−3^) day^−1^ and this coincided with the detection of *Methanoculleus* sp. (70.5%), *Methanosarcina* sp. (16.6%), and *Methanomasiliicoccus* sp. (11.6%). Hydrogen consumption could still be detected within the pH 10.0 enrichments; however, the rate fell from that observed at pH 7.0 [43.1 (±6.4) μmol day^−1^] to 17.4 (±4.6) μmol day^−1^. At both pH 7.0 and 10.0, *Methanoculleus* sp. dominated the community composition, representing 61.6 and 89.8% of the communities, respectively.

The pH of the Steel sediments ranged from pH 9.5 to 12.9. The enrichment of these sediments with either acetate or H_2_:CO_2_ demonstrated similar behavior to that observed with the New Lime sediments, again no acetate consumption was detected at pH 10.0 but was observed at pH 7.0 with a rate of 36.5 (±2.0) μmol day^−1^. At pH 7.0, acetoclastic communities from the Steel sites were dominated by *Methanosarcina* sp. (78.8%) with *Methanobacterium* sp. comprising 20.6% of the community. In contrast to the New Lime enrichments (Figure 3A), the Steel site sediments (Figure 5) demonstrated a greater rate of hydrogen consumption at pH 7.0 (90.8 ± 1.9 μmol day^−1^) than at pH 10.0 (85.1 ± 3.2 μmol day^−1^), although these were not significantly different (*p* = 0.105). The hydrogenotrophic methanogenic microcosms were again dominated by *Methanobacterium* sp.

Overall the results indicate that hydrogenotrophic methanogenesis is favored over acetoclastic methanogenesis at alkaline pH (>9.0). This observation is consistent across a wide range of calcium based anthropogenic sites of different origins and ages. Under the prevailing geochemical conditions created at these pH values the acetoclastic methanogenic pathway is unable to access its substrate due to the absence of undissociated acetic acid at these pH values.

## Discussion

The data presented here suggest that an alkaline pH >9.0 results in a methanogen community that is dominated by hydrogenotrophs, with populations enhanced by the activity of SAO bacteria. The methanogen populations from high pH and neutral sediments exposed to alkaline pH were both dominated by *Methanobacterium* sp., and in the case of the New Lime sediment communities, were capable of hydrogenotrophic methanogenesis at pH 11.0. Members of the Methanobacteriales have also been detected within a range of hyperalkaline environments resulting from serpentinisation processes (Brazelton et al., 2017; Crespo-Medina et al., 2017). A number of alkaliphilic *Methanobacterium* sp. have been isolated to date (Boone et al., 1986; Mathrani et al., 1988; Kotelnikova et al., 1998), the observations made here suggest that this genus is adaptable to aggressive pH changes as observed with Control sediment enrichments, but is also capable of surviving high pH after long-term exposure as residents of the initial New Lime sediments. This is in contrast to the Methanomicrobiales such as *Methanocalculus* sp. which are more prevalent in the hypersaline-hyperalkaline Soda lakes (Surakasi et al., 2007; Antony et al., 2013; Sorokin et al., 2015), due to the low salt tolerance of *Methanobacterium* sp. (Boone et al., 1986). In this study, *Methanosarcina* sp. were most impacted in the control sediments exposed to high pH in acetate enrichments. Pure culture studies of Lonar lake *Methanosarcina* indicated a maximum pH of 9.5 for growth upon acetate (Thakker and Ranade, 2002), which suggests that this genus may require a saline rather than a calcium dominated environment to sustain methane generation above pH 9.0.

These observations indicate that the generation of methane through the acetoclastic pathway, although energetically favorable with respect to bicarbonate (Jin and Kirk, 2018) does not proceed under alkaline conditions (>pH 9.0). High pH favors the dissociation of acetic acid to its anion (CH3COO^−^), preventing transmembrane diffusion (Kröninger et al., 2014). Under alkaline conditions, acetate transport into the acetoclastic methanogen cell is therefore reliant on an acetate transporter, which is likely to be less energetically favorable than hydrogenotrophic methanogenesis (Kröninger et al., 2014). Much of the current understanding of methanogen ecology under alkaline conditions has been focused upon the microbiology of hypersaline, hyperalkaline Soda lakes, where methanogenesis is most commonly associated with the metabolism of C-1 compounds being released upon the biodegradation of Cyanobacterial mats (Jones et al., 1998; Sorokin et al., 2017). The current study indicates that methanogenesis in non-saline, calcium dominated alkaline environments should be considered as a different ecological niche. Within these environments, hydrogenotrophic methanogenesis is the most prevalent methanogenic pathway above pH 9.0. This observation is supported by the investigations of sediments from 13 individual sites ranging in age, origin and chemical composition. These observations suggest that under the alkaline conditions generated within a cement-based GDF, it is the hydrogenotrophic methane generation pathway that will be active rather than acetoclastic methanogenesis.

## Data Availability Statement

The data is deposited with the NCBI under Bioproject accession number PRJNA525260.

## Author Contributions

PH, SR, WM, and HG contributed to conception and design of the study. RW carried out the experimental work. PH, WM, HG, RW, and SR participated in the field work. SR, PH, and RW wrote the first draft. All authors contributed to manuscript revision, read, and approved the submitted version.

### Conflict of Interest

The authors declare that the research was conducted in the absence of any commercial or financial relationships that could be construed as a potential conflict of interest. RWM Ltd. had no influence on the design or execution of this study or the interpretation and reporting of the data reported in this paper.
