# Supplementary material for: Hydrogenotrophic Methanogenesis Under Alkaline Conditions
Source: Front Microbiol. 2020 Dec 3;11:614227. doi: 10.3389/fmicb.2020.614227 (PMC7744349; doi:10.3389/fmicb.2020.614227)
Supplement: Supplementary file 1 [file Data_Sheet_1.docx]

**Data Sheet 1**

| **Reagent** | **Chemical Formula** | **Concentration (g/L)** |
| --- | --- | --- |
| Potassium dihydrogen phosphate | KH_2_PO_4_ | 0.27 |
| Disodium hydrogen phosphate dodecahydrate | Na_2_HPO_4_⋅12H_2_O | 1.12 |
| Ammonium chloride | NH_4_Cl | 0.53 |
| Calcium chloride dihydrate | CaCl_2_⋅2H_2_O | 0.075 |
| Magnesium chloride hexahydrate | MgCl_2_⋅6H_2_O | 0.10 |
| Iron(II) chloride tetrahydrate | FeCl_2_⋅4H_2_O | 0.02 |
| Resazurin (oxygen indicator) |  | 0.001 |
| Disodium sulfide | Na_2_S⋅9H_2_O | 0.1 |
| Stock solution of trace elements (Table 2) |  | 10 ml |

**Table S1. Mineral media composition per litre.**

| **Reagent** | **Chemical Formula** | **Concentration (g/L)** |
| --- | --- | --- |
| Manganese chloride tetrahydrate | MnCl_2_⋅4H_2_O | 0.05 |
| Boric acid | H_3_BO_3_ | 0.005 |
| Zinc chloride | ZnCl_2_ | 0.005 |
| Copper chloride | CuCl_2_ | 0.003 |
| Disodium molybdate dihydrate | Na_2_MoO_4_⋅2H_2_O | 0.001 |
| Cobalt chloride hexahydrate | CoCl_2_⋅6H_2_O | 0.1 |
| Nickel chloride hexahydrate | NiCl_2_⋅6H_2_O | 0.01 |
| Disodium selenite | Na_2_SeO_3_ | 0.005 |
| Disodium tungstate | Na_2_WO_4_⋅2H_2_O | 0.002 |

**Table S2. Trace element solution composition per litre.**

**Study Sites**

The investigation involved sediments from three lime kiln waste sites (≈25 to ≈150 years old) (New Lime) which are discussed further in Charles et al. (2019). Two of these sites were in North Yorkshire close to the towns of Settle (Site H) and Skipton (Site T) the third is close to the town of Buxton (Site B) in the UK. The Control site used to provide a neutral pH sediment was the Aspley Goit an inland water way that connects the Huddersfield Narrow canal and the river Colne, Huddersfield UK (≈200 years old). Five upland field kiln (Johnson, 2008) sites (200 to 300 years old) (Old Lime, Sites LK1, LK2, LK3, LK4, LK5) were also investigated and these were situated in Upper Warfdale, North Yorkshire, UK. The four steel industry waste sites (5 to ≈30 years old) (Steel, Sites CW, CS, RC, SC) investigated are described by Mayes et al. (2008).

| Site | | pH | | |
| --- | --- | --- | --- | --- |
|  |  | ***In-situ*** | **Sediment** |  |
| Control | | 6.9 | 6.9 |  |
| New Lime | B | 13.0 | 12.4 |  |
|  | H | 12.8 | 11.5 |  |
|  | T | 12.5 | 12.2 |  |
| Old Lime | LK1 | NT | 7.5 |  |
|  | LK2 | NT | 7.8 |  |
|  | LK3 | NT | 7.6 |  |
|  | LK4 | NT | 7.9 |  |
|  | LK5 | NT | 7.3 |  |
| Steel | CW | 12.0 | 11.3 |  |
|  | CS | 9.5 | 9.0 |  |
|  | RC | 12.8 | 12.2 |  |
|  | SC | NT | 10.8 |  |

**Table S3. Soil and in-situ pH values of the sediments employed in the investigation.**

pH 10.0

pH

11.0

pH

12.0

New Lime Sediment

pH

9.0

pH

8.0

pH

7.0

pH 10.0

pH

10.0

Hydrogen

Enrichments

CDP Enrichments

Acetate Enrichments

pH

11.0

pH

12.0

pH

9.0

pH

8.0

pH

7.0

pH 7.0

Control Sediment

pH 7.0

pH

10.0

Hydrogen

Enrichments

CDP Enrichments

Acetate Enrichments

**Figure S1. Experimental Scheme**. The CDP, Hydrogen and Acetate Enrichments were developed in a sequential manner that diluted out chemical influence of the inoculating materials.

**Figure S2. Methane Generation at a Range of pH Values from CDP fed New Lime Enrichments (n=2).** Across the pH range investigated, CDP fed enrichments of the three New Lime sediments demonstrated different responses to changing pH with the oldest site (B) generating methane across the whole range, Site T from pH 8.0 to pH 10.0 and Site H pH 9.0 and 10.0.

**Figure S3. Hydrogen Consumption at a Range of pH Values from H_2_/CO_2_ fed New Lime Enrichments (n=2).** Across the pH range investigated the three New Lime sediments demonstrated different responses to changing pH with the oldest site (B) generating methane across the whole range, Site T from pH 7.0 to pH 10.0 and Site H pH 9.0 and 11.0.

**Figure S4. New Lime Enrichments Cultured in the Presence of Fluoromethane.** In the presence of the acetoclastic methanogen inhibitor fluoromethane there was no observable impact of the removal of acetate. This indicates that acetate removal was coupled to methane generation via syntrophic acetate oxidation.

**Figure S5: Control Enrichments Cultured in the Presence of Fluoromethane.** In the presence of the acetoclastic methanogen inhibitor fluoromethane the removal of acetate was inhibited. This indicates that in these enrichments acetate removal is directly coupled to methane generation.

**References**

Charles, C.J., Rout, S.P., Wormald, R., Laws, A.P., Jackson, B.R., Boxall, S.A., et al. (2019). In-Situ Biofilm Formation in Hyper Alkaline Environments. Geomicrobiol. J. 1-7. doi: 10.1080/01490451.2018.1564803.

Johnson, D. (2008). The Archaeology and Technology of Early-Modern Lime Burning in the Yorkshire Dales: Developing a Clamp Kiln Model. Ind. Archaeol. Rev. 30(2)**,** 127-143. doi: 10.1179/174581908X347346.

Mayes, W.M., Younger, P.L., and Aumônier, J. (2008). Hydrogeochemistry of Alkaline Steel Slag Leachates in the UK. Water Air Soil Pollut. 195(1)**,** 35-50. doi: 10.1007/s11270-008-9725-9.
